# Synthesis, modification, and chlorhexidine loading of polymer modified‐HM‐HAP particles: In vivo antibacterial analysis and cell cytotoxicity assessment

**DOI:** 10.1002/smo2.70069

**Published:** 2026-07-09

**Authors:** Farishta Shafiq, Chenyu Liu, Simiao Yu, Yongxin Pan, Min Ji, Qingzhao Shi, Weihong Qiao

**Affiliations:** ^1^ State Key Laboratory of Fine Chemicals School of Chemical Engineering Dalian University of Technology Dalian China; ^2^ Department of Chemistry Dalian University of Technology Dalian China; ^3^ Zhengzhou Tobacco Research Institute China National Tobacco Corporation Beijing China

**Keywords:** antibacterial activity, cell cytotoxicity, chlorhexidine, hollow hydroxyapatite, in vivo‐study, polyethylene glycol

## Abstract

Hydroxyapatite (HAP) is commonly known as an excellent biocompatible and a potential solution to be used in drug delivery applications. This paper examines the synthesis, modification, and loading of chlorhexidine (CHD) drug into hollow mesoporous hydroxyapatite (HM‐HAP) particles to increase the antibacterial efficacy for oral infections. HM‐HAP particles were synthesized and modified with different weight polymers that is, polyethylene glycol (PEG (1000, 2000, 4.6k)) and polyethyleneimine (PEI (1200, 1800)), to improve the surface properties and drug loading ability. Chlorhexidine was effectively loaded onto both unmodified and polymer‐modified HM‐HAP samples, achieving loading efficiencies up to 81.67% for HM‐HAP and 77.85% for PEG‐1000/HM‐HAP verified by UV‐vis analysis. The CHD‐loaded samples were then eventually incorporated into sodium polyacrylate (PAAS) gels to improve their sticking properties, hence enabling effective adherence to the treatment site and enhancing the local antibacterial effect. The antibacterial activity of both CHD‐loaded polymer‐modified HM‐HAP samples and CHD‐loaded polymer‐modified HM‐HAP gel samples was evaluated both in vitro and in vivo. In in vitro tests, PEI‐1200/HM‐HAP and PEG‐1000/HM‐HAP gels exhibited the largest inhibition zones of ∼1.6 cm against *Escherichia coli* and ∼1.5 cm against *Staphylococcus aureus*, respectively. In vivo study evaluated the antibacterial efficiency of the CHD‐loaded polymer modified HM‐HAP gel samples by isolating oral bacteria from mouse teeth, with Gel‐PEI‐1200/HM‐HAP@CHD and Gel‐PEG‐1000/HM‐HAP@CHD samples demonstrating superior antibacterial performance. These findings suggest that CHD‐loaded polymer‐modified HM‐HAP is a promising therapeutic drug delivery system for oral health applications, with PEI‐modified HM‐HAP exhibiting the slight quickest release and PEG‐modified HM‐HAP showing sustained activity.

## Graphical abstract

This study presents the synthesis of hollow mesoporous hydroxyapatite (HM‐HAP) particles modified with different weights PEG and PEI. Chlorhexidine was loaded onto the particles. SEM analysis confirmed no significant morphological changes. Antibacterial activity was tested in vitro and in vivo using mouse oral bacteria, demonstrating promising results.

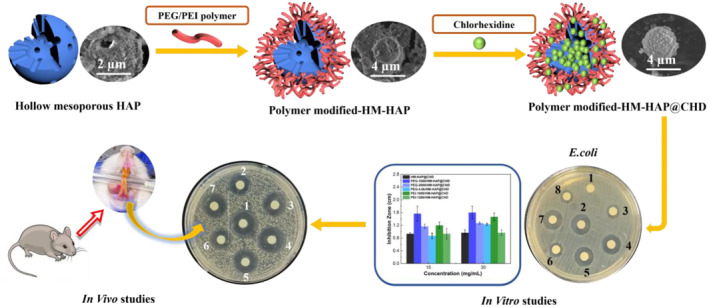

## 1 INTRODUCTION

Periodontal disease and dental caries are some of the dental infections that are widespread in the world and pose serious challenges to oral health.[^1^, ^2^] The medical therapy of these infections is mostly based on the usage of antibacterial agents like, chlorhexidine (CHD). CHD is known to be a broadly used antimicrobial agent in the treatment of oral care owing to its effectiveness against various forms of pathogens, especially against those linked with tooth plaque formation and periodontal diseases.^[3]^ However, there are numerous limitations related to the conventional drug delivery methods, often failing to maintain an effective antibacterial concentration at the infection site over prolonged durations and thus limiting their therapeutic efficiency.[^4^, ^5^, ^6^, ^7^] Consequently, this has led to an increase demand of the development of a novel drug delivery systems capable of providing sustained, localized release of antibacterial agents and improving the overall treatment of oral infections. The primary barrier to the provision of antibacterial drugs to the oral cavity is that these drugs are cleared out rapidly by saliva and the mechanical action of chewing, which shortens their retention duration and hence the therapeutic impacts.^[8]^ The already existing formulations, including mouthwashes or gels, do not tend to provide controlled and prolonged release at the targeted areas, which inhibits the effectiveness of bacterial removal over time.^[9]^ Thus, the need to introduce sophisticated delivery systems which assure sustained release, enhanced retention, and targeted antibacterial effect has become a key requirement to modern oral healthcare.

Hydroxyapatite (HAP, Ca_5_(PO_4_)_3_OH), a naturally occurring mineral form of calcium apatite, has become significant in biomaterials science owing to its biocompatibility, osteoconductivity, and structural resemblance to the mineral content of teeth and bone.[^10^, ^11^] HAP has found extensive applications in the field of bone tissue engineering, dental and drug delivery systems. Its ability to emulate the inorganic constituent of bone makes it a suitable option for regenerative medicine and targeted drug delivery systems.^[12]^ The HAP's porous framework allows drug adsorption that can aid in enhancing the concentration of the drug locally at the site of infection. Although hydroxyapatite is biocompatible, its surface characteristics are not optimal for effective drug loading and sustained release, which limits its ability to be efficiently used as a drug carrier in pharmaceutical applications.^[13]^

Historically, chlorhexidine (CHD) as an antimicrobial agent has been used as a key agent in the prevention and treatment of oral infections. It is extremely effective against a number of oral pathogens, including bacteria that cause tooth plaque and gingivitis.^[14]^ Regardless of its therapeutic importance, the swift elimination of CHD from the oral cavity remains a significant challenge, limiting its efficiency for long‐term usage. Therefore, the use of a drug delivery system that will allow the sustained release of chlorhexidine is of paramount importance in the maximization of its antibacterial activity and the ease of treating oral infections. To overcome these drawbacks, surface modifications of hydroxyapatite (HAP) with polymers such as polyethylene glycol (PEG) and polyethyleneimine (PEI) have been investigated for their biocompatibility and ability to impart favorable surface characteristics. PEGylation generally increases the hydrophilicity and biostability of HAP particles, while the addition of PEI introduction gives a positive charge to the surface, allowing the HAP to interact better with negatively charged therapeutic compounds like chlorhexidine (CHD).[^15^, ^16^] Both PEG and PEI‐modified inorganic particles have been investigated at the forefront concerning their capacity to express controlled drug release for applications in, wound healing, cancer treatment and bone regeneration etc..[^17^, ^18^, ^19^]

**FIGURE 1:**
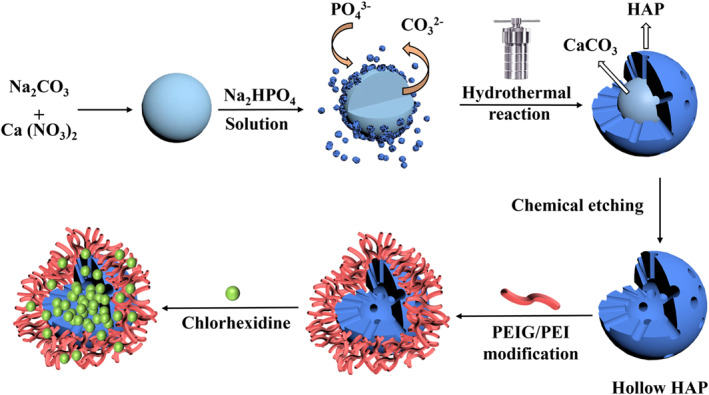
Diagram illustrating the synthesis procedure of PEG/PEI modified HM‐HAP@CHD particles.

The basic goal of this research project is to develop a biocompatible drug delivery system capable of delivering a sustained antibacterial effect in dental care. Particularly, this study is focused on the production of hollow mesoporous HAP (HM‐HAP) particles and its surface modification with PEG and PEI to stimulate the introduction and controlled release of the model antibacterial agent chlorhexidine. After that, the CHD‐loaded HM‐HAP particles are then embedded into sodium polyacrylate (PAAS) gels, which provide them with additional support in long‐term local retention and regulated release. The resulting formulations are subjected to test in the context of their antibacterial effect in relation to *Staphylococcus aureus* and *Escherichia coli*, which are prevalent pathogens in the infection of the oral cavity. Figure 1 illustrates the synthesis procedure of PEG/PEI modified HM‐HAP@CHD particles. Hence, this work highlights the potential of polymer‐modified HM‐HAP particles as a diverse and effective medium for the delivery of antibacterial drugs, providing sustained release and targeted therapeutic effects for the treatment of oral infections.

## 2 EXPERIMENTAL PROCEDURES

### 2.1 Materials and methods

Sodium chloride (NaCl), sodium carbonate (Na_2_CO_3_), potassium dihydrogen phosphate (KH_2_PO_4_), sodium hydroxide (NaOH), Agar, disodium hydrogen phosphate dodecahydrate (Na_2_HPO_4_·12H_2_O), calcium nitrate (Ca(NO_3_)_2_), and potassium chloride (KCl) were purchased from DAMAO, while poly(ethylene glycol) (PEG‐1000, PEG‐2000, PEG‐4.6k), polyethyleneimine (PEI‐1200, PEI‐1800), and sodium poly (styrene sulfonate) (PSS, Mw = 70,000) from Sigma‐Aldrich. Chlorhexidine and sodium polyacrylate (PAAS) were purchased from Aladdin, and LB broth from Qingdao Hope Bio‐Technology Co., Ltd. L292 cells were obtained from mouse fibroblast‐like epithelial cells acquired from American type culture collection. Dulbecco's modified Eagle's medium (DMEM), and fetal bovine serum (FBS) were obtained from Thermo Fisher Scientific. MTT assays were bought from KGI Biotech. 96‐well plates, and cell culture flasks were bought from Guangzhou Jet Bio‐Filtration Co., Ltd.

### 2.2 Synthesis and modification of HM‐HAP with different weight PEG and PEI polymers

The hydrothermal method was employed for the synthesis of hollow mesoporous hydroxyapatite (HM‐HAP) particles. This method initially started by rapidly mixing 20 mL of 0.1 M Na_2_CO_3_ solution with 20 mL of 0.1 M Ca(NO_3_)_2_ solution having 150 mg of PSS, accompanied by magnetic stirring at 25°C for 40 min. This consequently produced CaCO_3_ particles through a fast precipitation process, which were washed several times with deionized water and then collected using centrifugation process at 8000 rpm for 10 min. Afterwards, the CaCO_3_ particles were immersed in 30 mL of 0.8 M Na_2_HPO_4_ solution and subjected to hydrothermal treatment in an autoclave at 140°C for 6 h. Following the reaction, an etching step was conducted by introducing few drops of acid into the solution and stirring for 3 h to dissolve the CaCO_3_ core.

The synthesized HM‐HAP particles were then modified by PEG and PEI polymers. For this purpose, the HM‐HAP particles were mixed with PEG (molecular weights: 1000, 2000, and 4600 Da), and PEI (molecular weights: 1200, 1800 Da) at a mass ratio of 1:1 and stirred overnight (400 rpm) at room temperature. A smaller concentration of PEG was favored to prevent the entanglement of particles with a more substantial PEG and PEI layer. The PEG and PEI modified particles were then centrifuged at 10,000 rpm for 10 min, washed with distilled water and freeze dried.

### 2.3 Loading of chlorhexidine (CHD) drug

Chlorhexidine (CHD) drug was loaded into PEG and PEI‐modified hydroxyapatite in the following manner. First, a solution of 0.1% CHD was prepared. A specific volume of this solution was then taken and 500 mg of each sample was added separately for each individual experiment. The mixtures were afterwards stirred at room temperature overnight. Later, the samples were filtered and rinsed with distilled water to get rid of any unattached drug. The samples were then put in an oven to get dried and obtained the CHD‐loaded modified HM‐HAP labeled as PEG/PEI‐HM‐HAP@CHD. The washed filtrate was analyzed using UV spectrophotometry at λmax = 254 nm, and the loaded drug amount in each sample was determined using the following Equation (1):

(1) $$\%\;\text{Drug}\;\text{loading}=\frac{A-B}{A}\times 100$$

where “A” and “B” represent the initial and final drug concentrations of the drug solution.^[20]^

### 2.4 Preparation of sodium polyacrylate gel incorporating CHD drug loaded PEG and PEI‐modified HM‐HAP

1 g of sodium polyacrylate was used to make the polymer matrix for the gels. To this, 10% (w/w) deionized water was added, and the mixture was agitated with an overhead stirrer (German WIGGENS, WB2000‐M) at 400 rpm for 1 h at room temperature (25°C) until it became a uniform solution. Chlorhexidine‐loaded PEG or PEI‐modified hydroxyapatite (HAP) powders were then added in different concentrations of 1%, 5%, and 10% w/w relative to the PAAS gel. The powders were mixed thoroughly for 30 min using the overhead stirrer to achieve uniform dispersion of the particles through the PAAS gel. The formed gels incorporated with the CHD loaded samples were immediately frozen using liquid nitrogen and subsequently freeze‐dried under the following conditions: temperature = −30°C at the primary stages followed by secondary drying at 30°C, pressure = 0.05–0.01 mbar, and time = 48 h to obtain the porous final product.

### 2.5 In vitro study‐CHD drug release

In vitro release of CHD from PEG and PEI‐modified HM‐HAP was performed at 37°C in PBS (pH 7.4). CHD‐loaded HM‐HAP at 50 mg was immersed in the 30 mL of PBS release medium, which was maintained in a water bath shaker at 37°C with continuous agitation at 300 rpm. At predetermined time intervals that is, 1, 2, 3, 4, 5, 6, 8, 10, and 12 h, 3 mL buffer solution was obtained using a micro pipette for UV‐vis analysis. After which the withdrawn sample was immediately replaced with the same volume of fresh PBS buffer solution in to the system. The collected samples were then analyzed using UV‐vis spectroscopy at wavelength λ = 254 nm. The content of CHD was calculated via a standard curve and these analyses were repeated three times, and the results are reported as mean values.

(2) $$\text{Cumulative}\;\text{release}\;\text{of}\;\text{CHD}=\frac{C_{t}}{C_{0}}\times 100\%$$

Here, “*C*_*t*_” is the cumulative release concentration of CHD after time “*t*”, and “*C*_0_” is the initial concentration of CHD loaded in the particles.

### 2.6 Cell viability and cell culture

To check the cell viability of the synthesized samples, L929 cells were used. L929 cells were derived from mice and grown in DMEM medium enriched with 10% FBS and 1% antibody. The cell culture flasks T75 were used to culture the cells. The cell culture was maintained in a 37°C incubator with 5% CO_2_ and was replaced with fresh DMEM medium daily to promote the exponential growth of the cells. Each day, cells were washed 3 times with PBS to remove any residual medium or dead cells and this process was continued for 1 week until 80% confluence was achieved. After that, the L292 cells were plated in 96‐well plates and incubated at 37°C with 5% CO_2_ until the cell density reached 80%–90%. Then, fresh medium with different concentrations of CHD‐loaded samples was added to the 96‐well plates and incubated for 24 h. The cytotoxicity of CHD‐loaded HM‐HAP samples on L929 cells was assessed using the MTT assay. 10 μL of CCk‐8 was then added to each well and placed for roughly about 4 h to incubate. Blank medium was used as the control group, and the absorbance at 450 nm was measured to calculate the cell viability.

### 2.7 In vitro antibacterial activity tests

The antibacterial activity of the synthesized unmodified HM‐HAP@CHD and PEG and PEI‐modified HM‐HAP@CHD samples was assessed by applying agar diffusion technique. Sterile paper discs served as carriers for the sample materials. *E*. *coli* and *S*. *aureus* were chosen as demonstrative gram‐negative and gram‐positive bacteria. For performing the antibacterial tests, solid medium plates were prepared initially by mixing agar (0.75 g), and LB broth (1.25 g). The mixture was then sterilized using an autoclave. The sterilized medium was then added to sterile petri dishes and allowed to cool and solidify at room temperature before use. Sterile PBS buffer solution was used to dilute the bacterial concentration of 10^5^ CFU/mL. Afterward, 100 μL of each diluted bacterial solution was equally dispersed over the surface of the sterile LB agar medium. Prior to starting the experiment, the sterile paper discs were dipped into the sample solutions, allowed to absorb the solution sufficiently and then positioned on the surface of the LB agar medium. The discs were gently squeezed using sterile forceps to ensure sufficient interaction with the medium. The plates were then put in an oven and kept 37°C for 24 h. After the incubation period, inhibition zones were measured and photographed, and their diameters were measured. Each experiment with varying concentrations for each sample was conducted three times and the average was calculated.

All studies with the sodium polyacrylate (PAAS) gel formulations followed the same disc‐diffusion method as described for *E*. *coli* and *S*. *aureus*. The primary distinction was that rather than using standard laboratory strains, bacteria were collected and tested directly isolated from the mouse oral cavity to determine the efficiency against therapeutically relevant microbial communities.

### 2.8 In vivo studies

The animal procedures were authorized by the Dalian University of Technology Biomedical and Medical Ethics Committee having the approval number DUTSCE250612‐01 and were carried out in alignment with the institutional guidelines. Adult mice weighing 20–24 g were anesthetized via intraperitoneal injection of a freshly made working solution of tribromoethanol (Avertin) that is, 1 g tribromoethanol was dissolved in 45 mL sterile 0.9% NaCl. Each mouse was given 200 μL of the prepared working solution ∼220 mg/kg, resulting in fast induction within 1 min and establishing a surgical level of anesthesia for the procedure's length. The mice were kept on a warmed surface and observed until recovery. Following the induction of anesthesia, the oral cavity was exposed and a commercially available plaque‐disclosing agent (pink stain) was spread on the teeth for 30 s to visualize areas of bacterial colonization. In clinical and experimental oral trials, plaque‐disclosing chemicals have served as a reliable method to locate biofilms.

Seven treatment sets (groups) were evaluated: (1) control hydrogel (no drug), (2) Gel‐HM‐HAP loaded with chlorhexidine (HM‐HAP@CHD), (3) Gel‐PEG‐1000/HMHAP@CHD, (4) Gel‐PEG‐2000/HM‐HAP@CHD, (5) Gel‐PEG‐4.6k/HM‐HAP@CHD, (6) Gel‐PEI‐1800/HM‐HAP@CHD, and (7) Gel‐PEI‐1200/HM‐HAP@CHD. The test formulation for each group comprised the specified HM‐HAP particles incorporated into the pre‐prepared PAAS gel carrier. For each animal, the stained tooth areas were treated by putting 1 g of the appropriate PAAS gel formulation; containing the appropriate particle type and concentration onto the stained tooth surface with a sterile micro brush so that the material covered the visually identifiable bacterial area. The material remained in contact with the tooth for 1 h under anesthesia to allow interaction and initial drug release, following with the oral cavity was gently rinsed with distilled water to remove excess gel. After the exposure period, the plaque‐disclosing agent was applied again for 30 s and washed off to visualize the residual stained regions. High‐resolution images of the tooth surfaces were captured under uniform lighting settings before the treatment (after initial staining), and immediately following post‐treatment rinse as required by the study.

### 2.9 Characterizations

The HITACHI‐SU8220 scanning electron microscope (High‐Tech Corporation) was employed to analyze the modified and unmodified material's surface morphology and size. The X‐ray diffraction (XRD) measurements were examined using a Smart lab X‐ray diffractometer (Rigaku corporation) with diffraction angles spanning from 10 to 60° of 2*θ* to ascertain the crystal structure of the synthesized materials. BET‐surface area, pore diameter, and pore structure of the samples was assessed via Brunauer‐Emmett‐Teller (BET) analysis, carried out by an accelerated surface area porosimetry system (model no. ASAP‐2425). The FTIR spectra of the material were acquired using the KBr pellet technique with a ThermoFisher 6700 FTIR spectrometer. The rheological properties of the synthesized sodium polyacrylate (PAAS) gel were determined by an advanced Rheometer (TA Instruments, USA, model no. AR2000ex). Spectroscopic analysis was conducted utilizing a UV‐vis spectrophotometer carry‐100 (Agilent) was used to measure the absorbance of CHD drug throughout the study. The zeta potential of the produced materials was assessed using the Zetasizer Nano ZS 90 from Malvern, UK.

## 3 RESULTS AND DISCUSSION

### 3.1 Characterization of unmodified and polymer modified HM‐HAP

The current work involved the synthesis and modification of HM‐HAP particles with different weight PEG and PEI polymers. The HM‐HAP particles were first synthesized using a hydrothermal technique utilizing polystyrene sulfonate (PSS) as a crystal growth enhancer, followed by etching with acid in order to remove the calcium carbonate core to obtain a hollow structure. After synthesis, the particles were modified using PEG (PEG‐1000, PEG‐2000, PEG‐4.6k) and PEI (PEI‐1800, PEI‐1200) by immersing HM‐HAP particles in the appropriate polymer solutions under controlled experimental conditions. The modified HM‐HAP particles were then loaded with chlorhexidine (CHD) via mixing with CHD aqueous solution under stirring, followed by centrifugation and drying in an oven. The drug loading and release efficiency were eventually determined.

**FIGURE 2:**
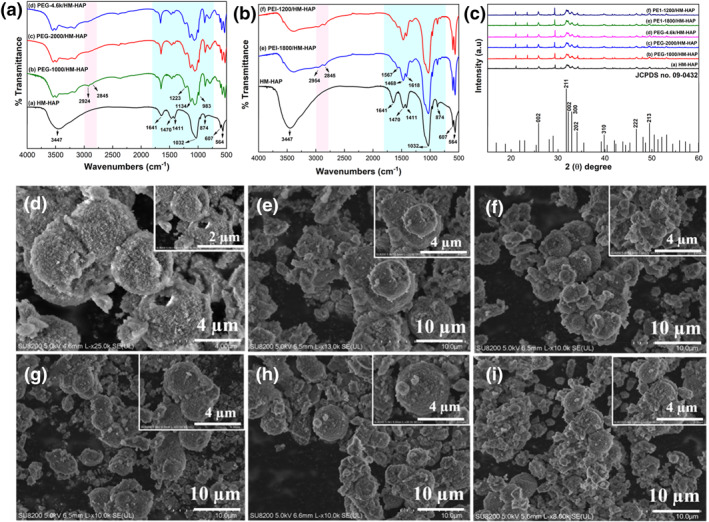
FTIR spectra (a, b), X‐ray diffraction analysis spectra (c) of unmodified and PEG/PEI‐modified HM‐HAP, and SEM micrographs of HM‐HAP (d), PEG‐1000/HM‐HAP (e), PEG‐2000/HM‐HAP (f), PEG‐4.6k/HM‐HAP (g), PEI‐1800/HM‐HAP (h), PEI‐1200/HM‐HAP samples.

The FTIR spectra of the synthesized hollow mesoporous hydroxyapatite (HM‐HAP) and its polymer‐modified samples were obtained to confirm the successful modification of HM‐HAP with different weight polymers that is PEG (1000, 2000, 4.6k) and PEI (1200, 1800). The FTIR spectrum of HM‐HAP (Figure 2a) displays characteristic peaks corresponding to the HAP structure, such as the broad peak at 3447 cm^−1^, which corresponds to the O‐H stretching vibration. The peak at 1641 cm^−1^ corresponds to H‐O‐H bending vibrations, displaying the presence of adsorbed water, whereas the peak at 1032 cm^−1^ is associated with the stretching mode of PO_4_^3‐^, confirming the phosphate structure of HAP. The typical peaks at 564 cm^−1^ and 607 cm^−1^ are ascribed to the bending vibration of PO_4_^3‐^. The distinctive B‐type CO_3_^2‐^ bands were positioned at 1466, 1410, and 873 cm^−1^.[^21^, ^22^]

The FTIR spectra of polymer‐modified HM‐HAP samples confirm the successful addition of both PEG and PEI onto the HM‐HAP surface. All PEG‐modified samples exhibited new characteristic peaks at 2845 cm^−1^ and 1223 cm^−1^ aligned with the C‐H stretching and C‐O‐C stretching vibrations of PEG, respectively.^[23]^ Similarly, PEI‐modified samples (Figure 2b) showed prominent C‐H stretching vibrations around 2954 cm^−1^ and 2845 cm^−1^ in conjunction with N‐H bending vibrations in the range 1411–1470 cm^−1^ verifying the successful grafting of PEI onto HM‐HAP.^[24]^ Notably, the absorption bands in the region 1000–1200 cm^−1^, characteristic of the phosphate groups (P‐O bond), are still present, demonstrating that the basic HAP structure remains intact even after modification with PEI. These data combined suggest that polymer functionalization was effective, happening predominantly on the surface without altering the interior HAP structure.

The XRD patterns of the as‐synthesized hollow mesoporous hydroxyapatite (HM‐HAP) and its polymer‐modified variants (Figure 2c) were assessed in order to evaluate their crystallinity and phase purity. The diffraction peaks observed in all samples showed that are characteristics of a hexagonal HAP structure. These peaks noted resembles the standard HAP diffraction data that is, JCPDS No. 09–0432, which shows that a pure, single‐phase hydroxyapatite has been formed.[^25^, ^26^] The retention of the distinctive HAP peaks in the polymer‐modified samples shows that the surface modification with PEG or PEI did not affect the crystalline structure of HAP.

For SEM imaging, the samples were firstly placed on conducting tape to ensure proper electrical conductivity during analysis. A thin, uniform layer of the sample was then spread on the tape using a pressure air dispersion device, which applied pressure to expel air and evenly distribute the sample. This method helps achieve a consistent and even coating. The SEM was operated at an accelerating voltage of 10–15 kV to achieve high‐quality resolution and clear surface analysis. The SEM images of the unmodified and polymer‐modified HM‐HAP samples displayed in Figure 2d–i provide information about the morphology and surface characteristics of the materials. The synthesized unmodified HM‐HAP (Figure 2d) had a uniform hollow mesoporous structure with well‐defined spherical shapes, confirming the effective synthesis of hollow mesoporous HAP. The overall hollow morphology of the particles was preserved even after modification with PEG and PEI. However, the polymer‐modified samples showed some particle agglomeration, which was probably caused by interactions between polymer chains and HAP surfaces during the modification process. In addition, the hollow properties were more evident in the unmodified HAP than in the modified samples, which is possibly because of the subtle surface alterations resulting from polymer functionalization. Altogether, the SEM analysis shows that the polymer modification does not alter the hollow spherical structure of HAP, while slight agglomeration is evident in the modified samples.

**FIGURE 3:**
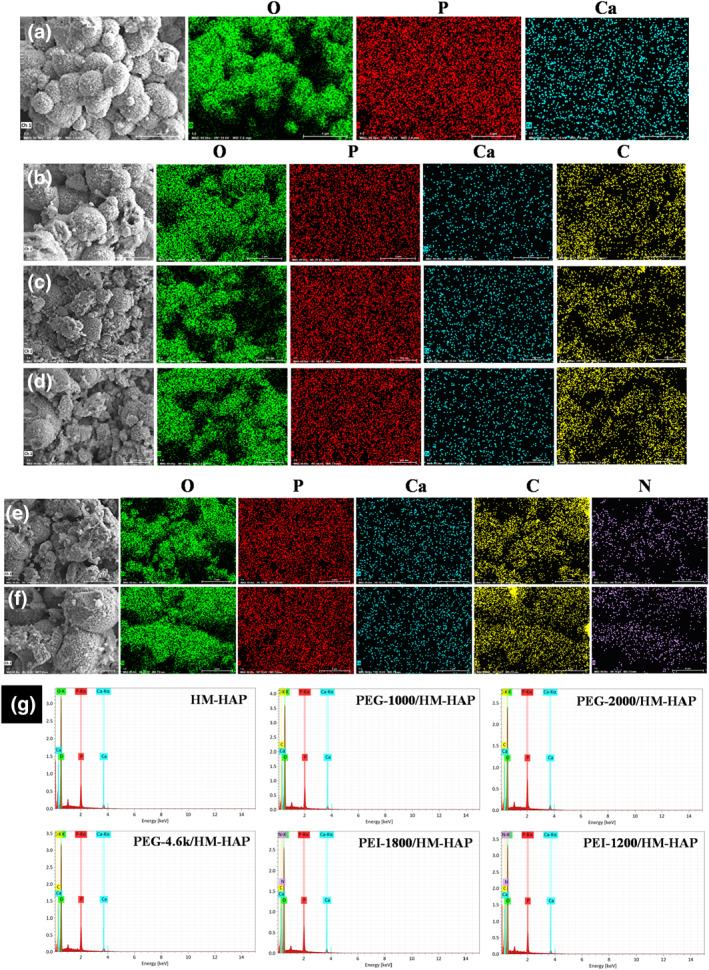
EDS elemental mapping (a–f), and EDS spectra (g) of the synthesized samples.

Following the SEM analysis, Energy Dispersive X‐ray Spectroscopy (EDS) was performed on the same samples to obtain the surface elemental composition and contents of unmodified HM‐HAP and PEG/PEI‐modified HM‐HAP samples. For the EDS analysis, the samples were scanned with the SEM under 20 kV accelerating voltage. The elemental mapping analysis (Figure 3a–f) for the unmodified HM‐HAP reveals that the sample has elements such as Ca, P, and O, which are the elements characteristic of an HAP structure. The elemental mapping profile of PEG‐modified HM‐HAP indicates the element C arising due to the PEG modification of the HAP surface besides the typical elements P and Ca. The distribution of C uniformly distributed over the surface shows the homogeneous modification of the HM‐HAP surface with PEG. In PEI‐modified HM‐HAP, the elemental mapping reveals that both C and N are present, which is indicative to the PEI modification, along with the characteristic peaks of P and Ca from HM‐HAP. The uniform distribution of the N and C on the surface demonstrates that the HM‐HAP surface was successfully modified with PEI. These can be further confirmed by the corresponding EDS spectra, as shown in Figure 3g, which shows clear C and N peaks in the PEG and PEI‐modified samples, respectively. These spectra also endorse the elemental composition and effective surface modification of HM‐HAP with PEG and PEI, and the constant presence of P and Ca elements, which are also characteristic of the HM‐HAP structure. The elemental mapping and EDS spectra results validate the effective modification of HM‐HAP with PEG and PEI as evidenced by the spatial distribution of their corresponding elements across the modified surfaces.

**FIGURE 4:**
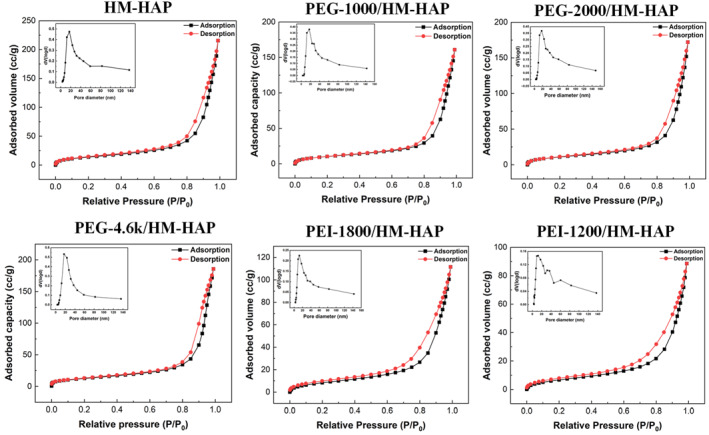
N_2_ adsorption‐desorption and corresponding pore size distribution graphs of unmodified and polymer‐modified HM‐HAP.

Following the validation of synthesis and modification by FTIR, XRD and SEM, which verified the integration of functional groups, crystallinity and morphology that is, uniform hollow mesoporous structure with slight agglomeration in modified samples, the BET surface area analysis was conducted to further assess the surface area and pore size distribution. The powdered samples were first degassed at 120°C for 12 h under vacuum to remove moisture or any other adsorbed volatile substances. The BET analysis was carried out at 77 K with nitrogen (N_2_) gas as the adsorbate. The Brunauer‐Emmett‐Teller (BET) specific surface area and pore size data for the HM‐HAP and PEG‐ and PEI‐modified HM‐HAP samples are detailed in Supporting Information S1; Table S1, whereas the nitrogen adsorption‐desorption isotherms and related pore size distribution graphs are demonstrated in Figure 4. On the basis of IUPAC classification, the results show that each of the samples has type IV isotherms with H3 hysteresis patterns. This proves the existence of mesoporous characteristics resulting from slit‐shaped pores formed by the aggregation of the particles.^[27]^ The unmodified HM‐HAP displayed a BET‐specific surface area of 68.719 m^2^/g and a pore size of 12.331 nm, presenting its pronounced porosity and hollow morphology. The modification with PEG resulted in reduction of the surface area compared to the unmodified hydroxyapatite (HM‐HAP) sample, likely because of the surface coverage effect. However, among the PEG‐modified samples, PEG 4.6k exhibited the highest surface area of 55.152 m^2^/g with a pore diameter of 17.281 nm, followed by PEG 2000 with 52.039 m^2^/g and PEG 1000 with 51.383 m^2^/g, presenting that with increase in molecular weight of PEG results in a greater surface area likely because of the larger PEG molecules creating more surface roughness and structural changes. Whereas, the PEI‐modified samples with PEI 1800 and PEI 1200 demonstrate lower surface areas (45.343 m^2^/g and 36.846 m^2^/g) possibly due to more surface coverage and pore blocking resulting from the branched structure of PEI. The pore diameter for PEG 4.6k was the largest with 17.281 nm, as larger PEG molecules extended the pore network, while PEI modifications resulted in smaller pores, demonstrating their role in restraining the pore structure. Hence, the BET surface area analysis provided valuable insights into the material's surface area and pore size distribution, validating the effect of polymer modification on the HM‐HAP's porosity.

Next, zeta potential analysis was conducted to determine the surface charge and stability of the modified and unmodified HM‐HAP samples. For this, 5 mg of the sample was dispersed into 50 mL of deionized water, followed by employing a probe sonicator (Ningbo science biotechnology Co., Ltd) to sonicate the mixture for 5 min to ensure homogeneous dispersion and to reduce agglomeration of the particles. After sonication, the sample was subjected to zeta potential measurements at room temperature. The zeta potential measurements of the unmodified and polymer‐modified HM‐HAP samples showed notable changes in surface charge upon modification with PEG and PEI as seen in Table 1. The unmodified HM‐HAP showed a negative zeta potential of −27.0 mV, arising mainly because of the surface hydroxyl groups, which impart a negative charge. Modification with PEG resulted a gradual decline in the negative surface charge with values of −15.8 mV, −9.16 mV, and −7.22 mV for PEG‐1000, PEG‐2000, and PEG‐4.6k, respectively. This shift towards neutrality can be due to the steric shielding of the negatively charged hydroxyl groups by the PEG chains. In contrast, PEI‐modified HAP exhibited a positive zeta potential, with +17.3 mV of PEI‐1800 and + 12.6 mV of PEI‐1200, owing to the protonated amine groups of PEI on the surface of the particle. The higher molecular weight PEI (PEI‐1800) results in a slightly higher positive charge than PEI‐1200, revealing the increased number of protonated amine groups. These results indicate that surface functionalization not only modifies the surface chemistry but also enables effective tuning of HAP surface charge, which is anticipated to effect drug loading efficiency, release kinetics, and interactions with bacterial or cell membranes.

**TABLE 1:** Zeta potential values of unmodified and polymer‐modified HM‐HAP samples.

Sample	Zeta potential (mV)
HM‐HAP	−27.0
PEG‐1000/HM‐HAP	−15.8
PEG‐2000/HM‐HAP	−9.16
PEG‐4.6k/HM‐HAP	−7.22
PEI‐1800/HM‐HAP	+17.3
PEI‐1200/HM‐HAP	+12.6

**FIGURE 5:**
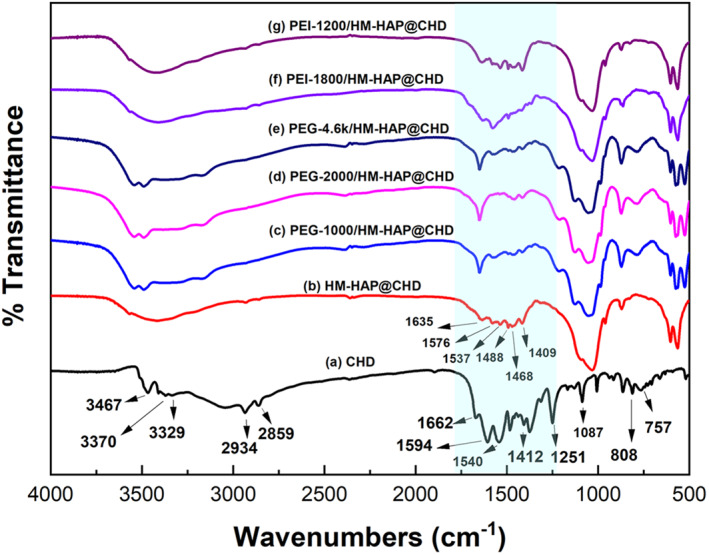
FTIR spectra of CHD loaded polymer‐modified HM‐HAP samples.

The synthesized HM‐HAP and polymer‐modified HM‐HAP samples were first thoroughly characterized to confirm their structural integrity and successful polymer modification. Afterward, chlorhexidine (CHD) was loaded into the unmodified and polymer‐modified HM‐HAP samples, and the successful incorporation of the drug was assessed by FTIR spectroscopy. The chemical structure of CHD is given in Supporting Information S1; Figure S1. The FTIR spectra (Figure 5) of chlorhexidine‐loaded HM‐HAP (HM‐HAP@CHD) and polymer‐modified samples confirm successful drug incorporation. Distinctive peaks of chlorhexidine, including C–Cl vibrations around 757 cm^−1^, C=N stretching near 1635 cm^−1^, and the C‐N stretching vibration of the imine group at 1662 cm^−1^, were detected in all drug‐loaded samples, confirming the existence of the drug on the HM‐HAP surface. In addition, the absorption bands at 1576 cm^−1^ and 1537 cm^−1^ are ascribed to the NH deformation vibrations of secondary amine and imine groups, further supporting the adsorption of chlorhexidine.[^28^, ^29^, ^30^] The broad stretching peak of O–H at about 3400 cm^−1^ and the P–O vibrations in the 1000‐1100 cm^−1^ region of HM‐HAP remained uninfluenced, affirming that the primary structure of the HM‐HAP was remined intact after the CHD drug loading. These spectral shifts indicate that CHD was successfully incorporated into the polymer‐modified HAP while maintaining its structural integrity. The SEM micrographs of all the CHD drug loaded samples are provided in Supporting Information S1; Figure S2.

**FIGURE 6:**
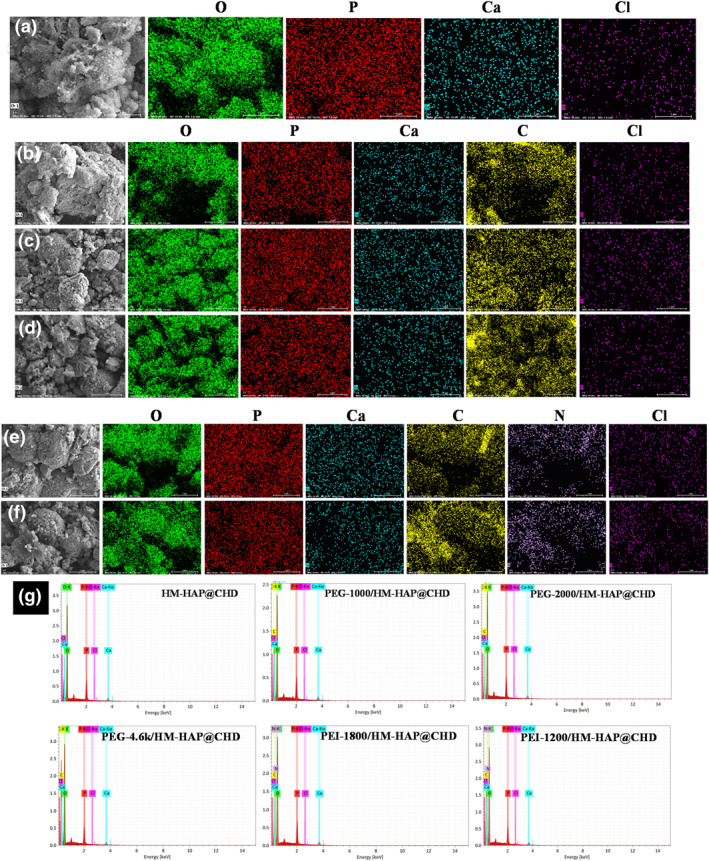
EDS spectra (a–f), and EDS spectra (b) of CHD loaded unmodified and polymer‐modified HM‐HAP samples.

The surface chemistry of the drug‐loaded materials was then investigated with the help of energy‐dispersive X‐ray spectroscopy (EDS). Figure 6a‐f shows the elemental mapping of all the CHD loaded HM‐HAP samples, which proves that there is a presence of “Cl,” which is representative of the CHD drug. Cl signal was uniformly observed over the surface of the particles, which indicates successful loading of the drug. This observation was consistent for all PEG and PEI‐modified HM‐HAP samples, revealing that CHD was effectively loaded into the HM‐HAP particles. These findings are further corroborated by the corresponding EDS spectra in Figure 6g where there are clear peaks of Cl along with the characteristic “P” and “Ca” elements arising from the HAP structure. The spectra also illustrate the C peak for PEG‐modified and PEI‐modified HM‐HAP samples, further validating the presence of PEG and PEI modifications, respectively. These findings confirm the successful loading of CHD into the modified HM‐HAP samples, with elemental mapping and EDS spectra offering clear evidence of the drug's incorporation into the HM‐HAP framework.

SEM micrographs of chlorhexidine‐loaded PEG and PEI‐modified HM‐HAP samples are shown in Supporting Information S1; Figure S2. As depicted in the images, drug‐loaded particles retain their original shape after loading with no large‐scale structural changes being observed. However, slight agglomeration of the particles is observed in some samples, which is typical during the drug‐loading process. This confirms the successful incorporation of chlorhexidine into the PEG and PEI‐modified HAP while maintaining the morphology of the particles.

The drug loading percentages for each sample were calculated using the calibration curve (Supporting Information S1; Figure S3) data for chlorhexidine, and the values are listed in Table 2. It can be seen that unmodified HM‐HAP displayed the highest drug loading at 81.67%, preceded by the PEG‐modified samples: PEG‐1000/HM‐HAP (77.85%), PEG‐2000/HM‐HAP (76.38%), and PEG‐4.6k/HM‐HAP (75.50%). The PEI‐modified samples showed comparatively lower drug loading with PEI‐1200/HM‐HAP at 52.62% and PEI‐1800/HM‐HAP at 68.74%. This trend in drug loading corresponds with the zeta potential data: the negatively charged HM‐HAP enables the highest drug loading owing to electrostatic attraction with the positively charged chlorhexidine, whereas the PEG‐modified samples, possessing reduced negative charge, demonstrates moderate drug loading through weaker electrostatic interactions and hydrophobic effects. The PEI‐modified samples, exhibiting positive zeta potentials, demonstrate lower drug loading attributed to the electrostatic repulsion between the positively charged PEI and positively charged chlorhexidine, hence reducing the drug adsorption potential.

**TABLE 2:** Table illustrating the % age drug loading of CHD in unmodified and polymer‐modified HM‐HAP samples.

Sample	Loading % age
HM‐HAP	81.67
PEG‐1000/HM‐HAP	77.85
PEG‐2000/HM‐HAP	76.38
PEG‐4.6k/HM‐HAP	75.50
PEI‐1800/HM‐HAP	52.62
PEI‐1200/HM‐HAP	68.74

### 3.2 In vitro release studies

**FIGURE 7:**
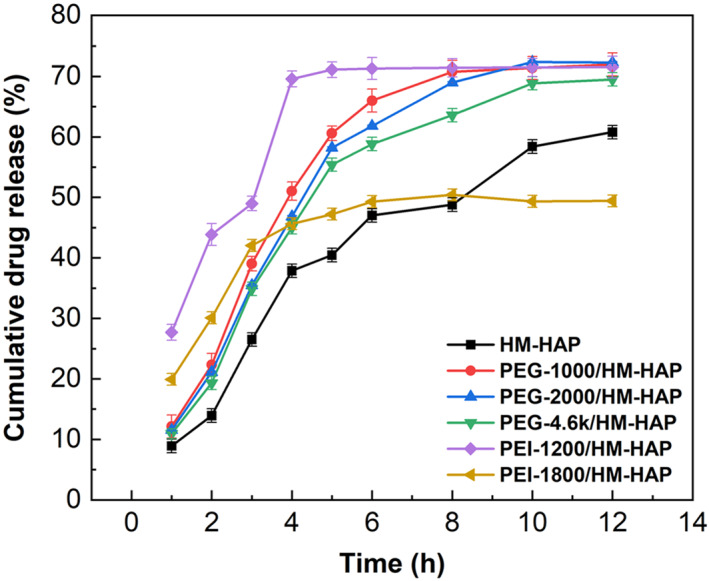
Drug release profile of CHD‐loaded unmodified and PEG/PEI‐modified HM‐HAP samples.

For in vitro release studies in order to mimic the chlorhexidine (CHD) release from CHD‐loaded PEG/PEI‐modified HM‐HAP, PBS buffer of pH 7.4 was employed to simulate the physiological conditions. Drug release tests were conducted by extracting 3 mL of the buffer at a specified time and afterward analyzed by UV spectrophotometer to determine the amount of drug released. The experiment was conducted three times, and the mean release profile was determined (Figure 7). It can be seen that cumulative drug release from all samples increased with time and the release profiles varied significantly with the type of polymer modification. Unmodified HM‐HAP showed the slowest release profile, achieving approximately 35% cumulative release after 12 h, possibly because of the strong electrostatic interactions between the negatively charged HAP surface and the positively charged CHD, which restricts drug diffusion. The drug release profiles of chlorhexidine‐loaded HM‐HAP samples, including unmodified and polymer‐modified HM‐HAP (PEG and PEI), were evaluated in PBS buffer at pH 7.4 to simulate physiological conditions. As shown in Figure 7, the drug release profiles varied significantly based on the type of polymer modification. Unmodified HM‐HAP exhibited the slowest release with a cumulative release of about 35% at 12 h. This gradual release is attained by the intense electrostatic bonds existing between the negatively charged surface of HM‐HAP and the positively charged chlorhexidine inhibiting the diffusion of drugs, thus inhibiting the release of the drug. PEG‐modified HM‐HAP samples demonstrated moderate release patterns with PEG‐1000/HM‐HAP showing the fastest release with nearly 60% release after 12 h. The reason could be possibly due to the smaller size of PEG‐1000 resulting in the less dense surface layer making the drug easier to diffuse. PEG‐2000/HM‐HAP had a moderate release profile, with approximately 50% release at 12 h, as the larger PEG size gives a more sterically shielded surface, thereby inhibiting the diffusion of CHD. PEG‐4.6 k HM‐HAP exhibited the slowest release rate of all the PEG‐modified samples with approximately 40% release at 12 h owing to the elevated molecular weight of PEG, which creates a denser surface coating and steric shielding, therefore, limiting the diffusion of drugs.[^31^, ^32^, ^33^] The PEI‐modified HM‐HAP samples exhibited the fastest drug release overall. PEI‐1200/HM‐HAP recorded a release of more than 70%, and PEI‐1800/HM‐HAP recorded a slightly low release of approximately 60% after 12 h. This high‐rate release of PEI‐modified samples could be attributed to the positive charge on PEI that caused the electrostatic repulsion with the positively charged chlorhexidine thereby increasing the rate of drug diffusion. Notably, PEI‐1200, possessing lower molecular weight, displayed a faster release than PEI‐1800 owing to its less dense polymer layer enabling for more rapid drug release.

### 3.3 Cell viability

**FIGURE 8:**
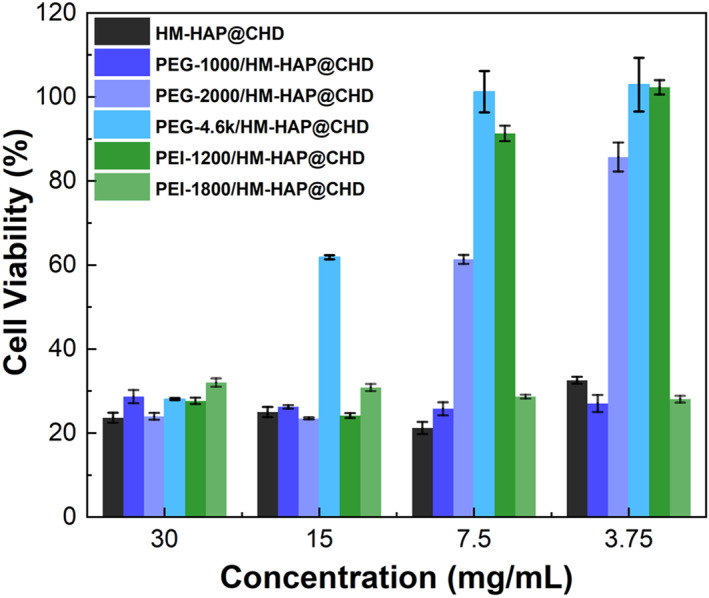
L929 cell viability of the unmodified and polymer‐modified HM‐HAP samples.

The cell viability of the CHD‐loaded HM‐HAP samples, including unmodified HM‐HAP and polymer‐modified HAP, was evaluated at different concentrations, including 30, 15, 7.5, and 3.75 mg/mL to evaluate their cytotoxicity. The findings presented in Figure 8 revealed that there were observable cytotoxicity differences depending on the polymer modification. At all the concentrations, unmodified HM‐HAP exhibited low cell viability with a maximum value reaching up to 25%. PEG/PEI modified HM‐HAP samples demonstrated to be more viable to the cell especially at lower concentrations. The PEG‐1000/HM‐HAP stood out as the least viable of the PEG‐modified HM‐HAP samples, likely because it had a smaller molecular size that allowed it to interact slightly more effectively with cell membranes. PEG‐2000/HM‐HAP and PEG‐4.6k/HM‐HAP showed favorable cell viability, with PEG‐4.6k/HM‐HAP recording the highest viability. This implies that longer PEG chains have better steric shielding and less direct cellular contacts, which leads to lower cytotoxicity.[^34^, ^35^, ^36^] PEG‐modified samples showed moderate declines in viability at higher concentrations (15 mg/mL and 30 mg/mL) with values ranging from 30% to 60%. PEI‐modified HM‐HAP samples, specifically PEI‐1800/HM‐HAP, demonstrated the lowest cell viability across all concentrations, with a significant decrease in cell viability to lower than 50% in 15 mg/mL and 30 mg/mL. The positively charged PEI polymer strongly binds with cell membranes, strongly resulting in membrane disruption and cytotoxicity. Furthermore, PEI‐1200/HM‐HAP showed slightly superior cell viability than PEI‐1800/HM‐HAP, presumably owing to the less dense polymer layer of PEI‐1200, which results in reduced cell interaction relative to PEI‐1800, which forms a more tightly bound surface. Hence, these results indicate that PEG‐modified HM‐HAP demonstrates superior biocompatibility and lower cytotoxicity compared to PEI‐modified HM‐HAP, with PEG‐4.6k showing the highest cell viability. PEI‐modified HM‐HAP, despite its increased cytotoxicity, may be more appropriate for applications prioritizing antibacterial characteristics over cell viability.

### 3.4 Antibacterial activity

**FIGURE 9:**
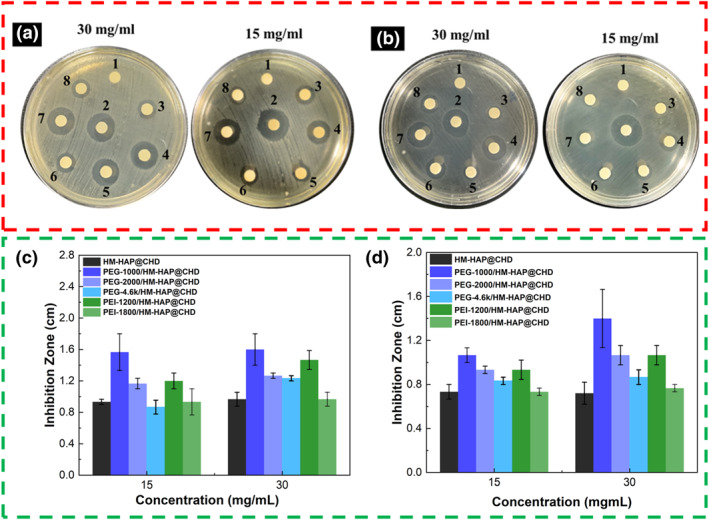
Analysis of antibacterial activity using paper disc method against (a) *E*. *coli*, (b) *S*. *aureus*, (c) graphical representation of antibacterial activity against (c) *E*.*coli*, and (d) *S*. *aureus*, illustrated by inhibition zone diameters from the graph.

The antibacterial activity of CHD‐loaded HM‐HAP samples, comprising unmodified HM‐HAP and PEG/PEI‐modified HM‐HAP, was assessed by measuring the inhibition zone against *E*. *coli* and *S*. *aureus* at concentrations of 15 and 30 mg/mL. In Figure 9, the samples labeled are as: (1) PBS (negative control), (2) CHD (positive control), (3) HM‐HAP@CHD, (4) PEG‐1000/HM‐HAP@CHD, (5) PEG‐2000/HM‐HAP@CHD, (6) PEG‐4.6k/HM‐HAP@CHD, (7) PEI‐1200/HM‐HAP@CHD, (8) PEI‐1800/HM‐HAP@CHD. Figure 9 shows that the PEI‐modified HM‐HAP samples, particularly PEI‐1200/HM‐HAP (7), displayed the largest inhibition zones for both bacterial strains and concentrations, signifying the highest antibacterial activity. The enhanced activity can be because of the positively charged PEI polymer, which promotes rapid drug release and strong electrostatic interactions with the negatively charged bacterial membranes, resulting in membrane disruption and effective bacterial inhibition. PEI‐1200/HM‐HAP which had a low molecular weight had a bigger inhibition zone compared to PEI‐1800/HM‐HAP. This is most likely due to the less dense polymer layer of PEI‐1200 that can facilitate faster release of drugs and more immediate antibacterial effects. On the other hand, PEG‐modified HM‐HAP samples were characterized with moderate antibacterial activity as compared to PEI‐modified samples.[^37^, ^38^] PEG‐1000/HM‐HAP had the largest inhibition zone among the various PEG molecules, proposing that smaller PEG chains results in faster CHD release and a more effective antibacterial activity. However, PEG‐2000/HM‐HAP and PEG‐4.6k/HM‐HAP exhibited smaller inhibition zones, with PEG‐4.6k/HM‐HAP demonstrating the least effective antibacterial activity. This trend may be attributed to the fact that larger molecular weight of PEG results in increased steric shielding and slower release of CHD, thus reducing the overall antibacterial efficiency. The smaller PEG chains in PEG‐1000/HM‐HAP result in a less shielded surface, allowing for faster drug diffusion and thus stronger bacterial inhibition.[^39^, ^40^, ^41^] It can be seen that the unmodified HM‐HAP also demonstrated antibacterial activity, but the inhibition zones were smaller relative to the PEG and PEI‐modified HM‐HAP samples (Figure 9). This is primarily due to the retarded release of CHD of the unmodified HM‐HAP. The HM‐HAP is negatively charged, which forms strong electrostatic interactions with the positively charged CHD resulting in stronger binding between these two, which subsequently slows down the release of the drug off the surface. Although these effective interactions may enhance the drug retention, the slower release of drug results in less immediate antibacterial activity, as the drug is released more steadily over time. Whereas, PEG and PEI‐modified HM‐HAP displayed larger inhibition zones because of their ability to facilitate drug release, which leads to faster antibacterial activities. In conclusion, it was demonstrated that PEI‐modified HM‐HAP samples, especially PEI‐1200/HM‐HAP, exhibit the strongest antibacterial properties due to the quicker release of CHD and higher electrostatic interactions with bacterial membranes. In contrast, PEG‐modified HM‐HAP samples are effective but provide more controlled release and moderate antibacterial activity, with PEG‐1000/HM‐HAP showing the optimal performance within the PEG group. These results indicate that PEI‐modified HM‐HAP is more appropriate for applications necessitating rapid antibacterial activity, where PEG‐modified HM‐HAP may be favored for sustained antibacterial activities.

### 3.5 Gel studies

After the successful synthesis of CHD‐loaded PEG/PEI‐modified HM‐HAP samples, the next phase included the incorporation of the CHD‐loaded PEG/PEI‐modified HM‐HAP samples into a sodium polyacrylate (PAAS) gel matrix. Sodium polyacrylate was chosen due to its ability to form a viscous, biocompatible gel. The rheological properties of the gel are described in Supporting Information S1; Figure S4. Three different compositions were prepared, incorporating PEG‐modified HM‐HAP@CHD, PEI‐modified HM‐HAP@CHD, and unmodified HM‐HAP@CHD into the PAAS gel matrix at different concentrations that is, 1%, 5%, and 10%. Post‐preparation, the PAAS gels were underwent through FTIR and SEM characterizations to confirm the successful incorporation of the HM‐HAP samples into the PAAS gel matrix and to analyze any structural alterations caused by the incorporation process.

**FIGURE 10:**
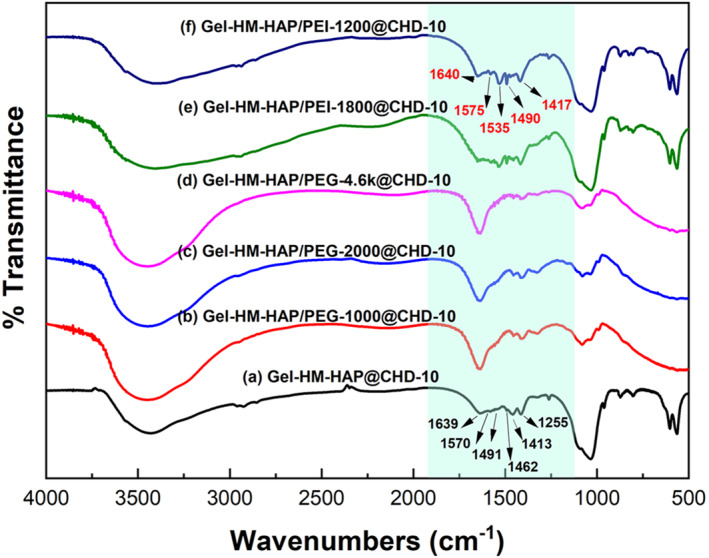
FTIR spectra of 10% CHD‐loaded polymer‐modified HM‐HAP incorporated into sodium polyacrylate gels.

Following the incorporation of CHD‐loaded PEG/PEI‐modified HM‐HAP samples into sodium polyacrylate gels, the FTIR spectra of the 10% CHD‐loaded PAAS gel samples in Figure 10 confirmed the successful incorporation of the samples into the gel matrix. The spectra indicate the characteristic absorption peaks of HM‐HAP and CHD, showing that the drug‐loaded particles are effectively incorporated into the gel. These findings confirm that the CHD‐loaded PEG/PEI‐modified HM‐HAP samples remains intact within the gel, assuring effective drug release and antibacterial potential. The FTIR spectra shown in Supporting Information S1; Figure S5 for 1% and 5% CHD‐loaded gel samples further supports the successful incorporation of the synthesized samples into the gel matrix.

**FIGURE 11:**
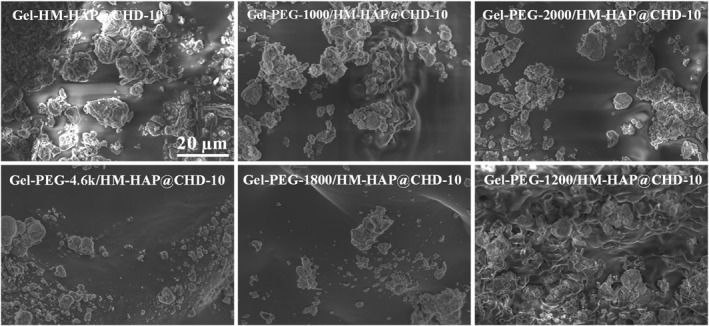
SEM micrographs of CHD‐loaded PEG/PEI‐modified HM‐HAP particles incorporated into the sodium polyacrylate gel matrix.

SEM images of CHD‐loaded HM‐HAP particles incorporated into sodium polyacrylate gels with various PEG and PEI modifications that is PEG‐1000, PEG‐2000, PEG‐4.6k, PEG‐1800 and PEG‐1200 are shown in Figure 11. The images clearly show the effective incorporation of all the sample's particles within the gel matrix without any significant changes in their morphology. The gel matrix is apparent encircling the particles signifying successful incorporation. Despite the modification, the particles maintained their natural structure, which confirms that the gel formulation does not alter the integrity of the HM‐HAP particles, rendering it acceptable for sustained drug delivery applications. Further SEM images at different magnifications are provided in Supporting Information S1; Figures S6 and S7, showing a more detailed view of the gel and particles.

**FIGURE 12:**
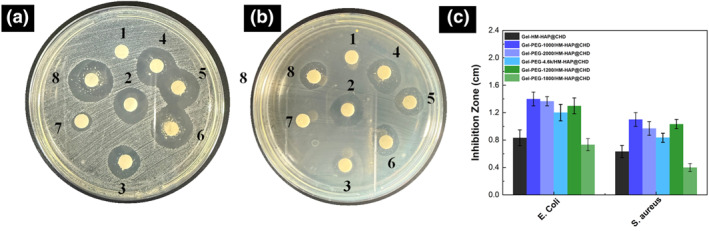
Analysis of antibacterial activity utilizing paper‐disc method against (a) *E*. *coli*, (b) *S*. *aureus*, (c) graphical illustration of antibacterial activity against *E*. *coli*, and *S*. *aureus* (c) represented by inhibition zone diameters.

Following the integration of the CHD‐loaded HM‐HAP samples into sodium polyacrylate gels and confirming their successful incorporation via FTIR and SEM characterizations, the antibacterial activity of the resulting gels was assessed using the similar inhibition zone method employed for the free CHD‐loaded HAP samples. The antibacterial activity of the respective samples is given in Figure 12. The samples are labeled as: (1) PBS (negative control), (2) CHD (positive control), (3) HM‐HAP@CHD, (4) PEG‐1000/HM‐HAP@CHD, (5) PEG‐2000/HM‐HAP@CHD, (6) PEG‐4.6k/HM‐HAP@CHD, (7) PEI‐1200/HM‐HAP@CHD, and (8) PEI‐1800/HM‐HAP@CHD, respectively. As illustrated in Figure 12a,b, the gels containing PEG‐modified HM‐HAP and PEI‐modified HM‐HAP demonstrated significant antibacterial activity against both *E*. *coli* and *S*. *aureus*, whereas that with the unmodified HM‐HAP displayed smaller inhibition zones, showing lower antibacterial effectiveness. Among the PEG‐modified gel samples, Gel‐PEG‐1000/HM‐HAP@CHD showed the largest inhibition zones, followed by Gel‐PEG‐2000/HM‐HAP@CHD and Gel‐PEG‐4.6k/HM‐HAP@CHD, consistent with the trend observed in the free gel samples. This is likely due to the faster drug release from the smaller PEG chains, which allows for more immediate antibacterial action. The Gel‐PEG‐4.6k/HM‐HAP@CHD had slightly reduced inhibition zones, which implies that CHD was released more slowly because of the larger size of PEG‐chains and greater steric shielding. The gel with unmodified HM‐HAP had the weakest antibacterial activity, which could be explained by the fact that chlorhexidine binds better to the negatively charged HM‐HAP and that the diffusion of the drug out of the gel structure occurred slower. In general, these findings indicate that the gel‐incorporated samples exhibit the same trends in patterns of antibacterial activities as detected in the free gel samples. PEI‐modified HM‐HAP gels demonstrated enhanced and more immediate antibacterial efficiency, whereas, PEG‐modified HAP gels showed moderate antibacterial activity, with lower PEG chains facilitating the faster drug release, and unmodified HM‐HAP gels showed reduced yet prolonged antibacterial effects. This validates that the incorporation of the drug‐loaded HM‐HAP into sodium polyacrylate gel does not affect the antibacterial activity while somewhat alters the release profile due to the gel matrix. In Supporting Information S1; Figure S8, the results for the 1% and 5% concentrations of the gels further support these trends, with Gel‐PEI‐1200/HM‐HAP@CHD and Gel‐PEG‐1000/HM‐HAP@CHD samples showing the enhanced antibacterial effects at these concentrations, while the 10% gels illustrated similar but slightly less pronounced activity.

### 3.6 In vivo studies

**FIGURE 13:**
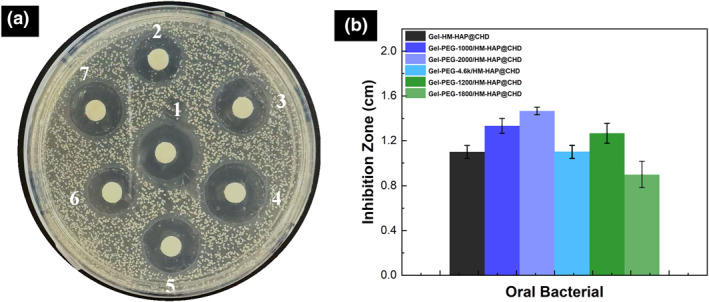
Analysis of antibacterial activity using the paper‐disc method against oral mouse bacteria (a), and graphical representation of antibacterial activity (b), represented by inhibition zone diameters.

To further analyze the antibacterial properties of the PAAS gel‐incorporated CHD‐loaded PEG/PEI modified HM‐HAP samples, oral bacteria was obtained from mouse teeth and gum regions, and the gels were applied to determine their activity against naturally occurring oral populations of bacteria. The experiment employed the identical inhibition zone method, and as illustrated in Figure 13, the gels incorporated samples showed significant antibacterial activity against the obtained oral bacteria. The samples are labeled as: (1) CHD (positive control), (2) HM‐HAP@CHD, (3) PEG‐1000/HM‐HAP@CHD, (4) PEG‐2000/HM‐HAP@CHD, (5) PEG‐4.6k/HM‐HAP@CHD, (6) PEI‐1800/HM‐HAP@CHD, and (7) PEI‐1200/HM‐HAP@CHD, respectively. Both PEG and PEI‐modified HM‐HAP gels displayed high levels of antibacterial activity, indicating that the addition of the drug‐loaded samples to the gel did not affect its functionality in a physiologically applicable environment. In particular, Gel‐PEG‐1000/HM‐HAP and Gel‐PEI‐1200/HM‐HAP exhibited selectively visible inhibition zones, indicating rapid drug release and efficient interaction with oral bacteria. These obtained results demonstrate that the gel formulations effectively inhibit the oral bacterial growth, validating the potential of CHD‐loaded PEG and PEI‐modified HM‐HAP gels for practical antibacterial applications in the oral cavity.

**FIGURE 14:**
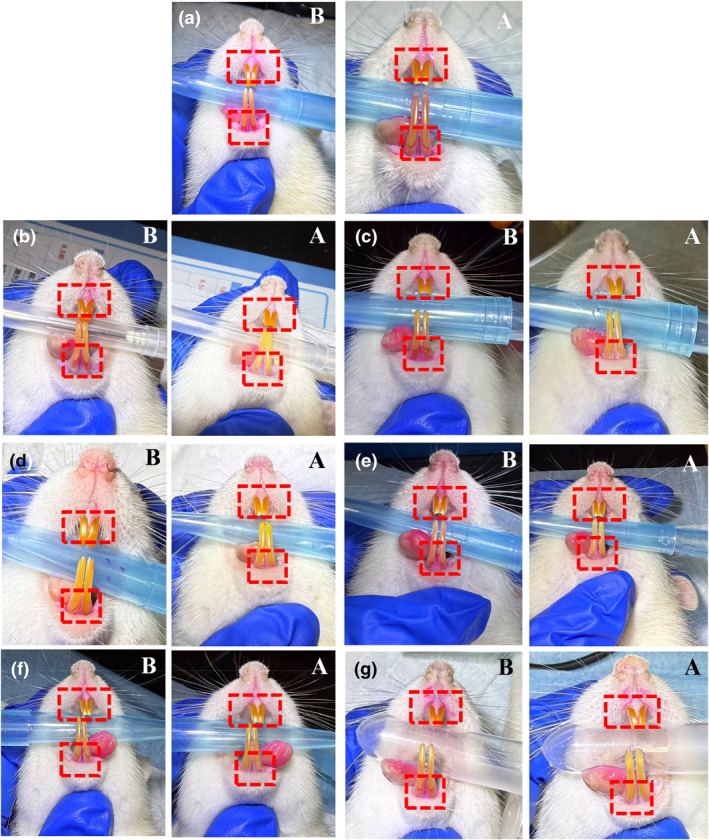
In vivo antibacterial activity of (a) PAAS gel (control), (b) gel‐HM‐HAP @CHD, (c) gel‐PEG‐1000/HM‐HAP@CHD, (d) gel‐PEG‐2000/HAP@CHD, (e) gel‐PEG‐4.6k/HM‐HAP@CHD, (f) gel‐PEI‐1800/HM‐HAP@CHD, (g) gel‐PEI‐1200/HM‐HAP@ CHD, showing the “before” (b) and “after” (a) treatment images of the stained tooth and gums surfaces in mice.

In vivo antibacterial efficiency of the CHD‐loaded HM‐HAP gel formulations was assessed in adult mice through the application of the various HM‐HAP‐based synthesized samples to stained tooth surfaces. The process starts with the use of a plaque‐disclosing agent to identify regions of bacterial colonization, then afterward followed by treatment with seven different groups of PAAS gel formulations. These groups included: (a) control Gel (no drug), (b) Gel‐HM‐HAP @CHD, (c) Gel‐PEG‐1000/HM‐HAP@CHD, (d) Gel‐PEG‐2000/HAP@CHD, (e) Gel‐PEG‐4.6k/HM‐HAP@CHD, (f) Gel‐PEI‐1800/HM‐HAP@CHD, and (g) Gel‐PEI‐1200/HM‐HAP@ CHD. Each gel formulation sample was gently applied to the stained tooth surface for 1 h under anesthesia, facilitating interaction and initial drug release. Following this treatment, the oral cavity was rinsed with water and images were taken. In Figure 14, the digital images clearly demonstrate the considerable difference between the “before” and “after” labeled as the (B) and (A) treatment states. In the control group (a), it can be seen that when only the PAAS gel was applied, the stained areas surrounding the gums remained persisted post‐rinsing, signifying that the gel alone did not have a noticeable impact on bacterial removal. It can be further verified by the antibacterial experiment only performed with the PAAS gel as shown in Supporting Information S1; Figure S8(a) with no observable antibacterial effect. On the other hand, all experimental groups, comprising those with CHD loaded HM‐HAP modified with different weight PEG and PEI polymers, demonstrated considerable decrease in bacterial presence. The digital images of these groups (A) of the after results exhibit little or no residual stain, which shows the efficiency of the antibacterial effect of the drug‐loaded HM‐HAP particles. These findings validate that both PEG and PEI‐modified HM‐HAP formulations possess remarkable antibacterial potential for the treatment of oral bacterial infections positioning them as potential options for localized drug delivery in oral healthcare applications.

## 4 CONCLUSION

In this research study, successful synthesis and characterization of hollow mesoporous hydroxyapatite (HM‐HAP) particles were carried out and afterward effectively modified with polyethylene glycol (PEG) and polyethylenimine (PEI) polymers. The resultant polymer‐modified HM‐HAP particles were then loaded with chlorhexidine (CHD) drug for enhanced antibacterial and therapeutic applications. The surface modification employing PEG and PEI markedly transformed the surface characteristics of HM‐HAP, resulting in improved drug release characteristics and enhanced antibacterial efficiency. The modification by PEG results in reduced surface negative charge of HM‐HAP leading to moderate drug loading and more controlled release, whereas PEI modification endowed the surface with a positive charge leading to swifter drug release and higher antibacterial activity. The incorporation of CHD‐loaded HAP into sodium polyacrylate gel was carried out to obtain the adhesive properties in order to adhere effectively to the treatment site and therefore enhance the local antibacterial effect. Cytotoxicity experiments conducted using L929 cells demonstrated that unmodified HM‐HAP exhibited comparatively low cell viability at all concentrations (30, 15, 7.5, and 3.75 mg/mL) with values lower than 50%. On the other hand, PEG‐modified samples, particularly PEG‐4.6k/HM‐HAP, illustrated better cell viability, with values ranging from 80% to 100% at lower concentrations, signifying reduced cytotoxicity relative to unmodified HM‐HAP. Nevertheless, PEI‐modified HM‐HAP samples especially PEI‐1800/HM‐HAP exhibited the lowest cell viability with values falling to below 40% at all the concentrations, which could be because of the strong interaction existing between the positively charged PEI polymer and cell membranes. Furthermore, the PAAS gels comprising CHD‐loaded PEG and PEI‐modified HM‐HAP samples displayed substantial antibacterial activity against oral pathogens, validating their potential for practical implementations in the field of oral healthcare. The present research indicates the versatility of polymer‐modified HM‐HAP as an efficient drug delivery system for targeted and prolonged antibacterial treatment with promising outcomes for the treatment of oral infections. Future research work can concentrate on further optimizing these formulations and assessing their in vivo performance for therapeutic applications.

## CONFLICT OF INTEREST STATEMENT

The authors declare no conflicts of interest.

## ETHICS STATEMENT

Ethical Approval for Animal Studies: All animal experiments were conducted in accordance with the ethical guidelines approved by the Biomedical and Medical Ethics Committee of Dalian University of Technology (approval number DUTSCE250612‐01) and in compliance with institutional regulations. Mice were housed under specific pathogen‐free conditions with ad libitum access to food and water. All experimental procedures were carried out with efforts to minimize animal suffering and in accordance with internationally accepted ethical standards for animal research. Ethical Approval for Cell Culture: L929 cells used in this study were obtained from ATCC and cultured in accordance with standard institutional biosafety and ethical guidelines for laboratory cell research. No human experiments were involved in this study.

## Supporting information

Supporting Information S1

## Data Availability

The data that support the findings of this study are available from the corresponding author upon reasonable request.
