# Supplementary material for: Synthesis, modification, and chlorhexidine loading of polymer modified‐HM‐HAP particles: In vivo antibacterial analysis and cell cytotoxicity assessment
Source: Smart Mol. 2026 Jul 9:e70069. Online ahead of print. doi: 10.1002/smo2.70069 (PMC13399578; doi:10.1002/smo2.70069)
Supplement: Supplementary file 1 — Supporting Information S1 [file SMO2-9999-0-s001.docx]

SUPPLEMENTARY INFORMATION

**Synthesis, Modification, and Chlorhexidine Loading of Polymer modified-HM-HAP Particles: In Vivo Antibacterial Analysis and Cell Cytotoxicity Assessment**

Farishta Shafiq ^a^, Chenyu Liu ^a^, Simiao Yu ^a^, Yongxin Pan ^a^, Min Ji ^b^, Qingzhao Shi ^c,^*, Weihong Qiao ^a,^*

^a^ State Key Laboratory of Fine Chemicals, School of Chemical Engineering, Dalian University of Technology, Dalian, 116024, P. R. China

^b^ Department of Chemistry, Dalian University of Technology, Dalian 116024, China

^c^ Zhengzhou Tobacco Research Institute, China National Tobacco corporation, P. R. China

* Corresponding author: qingzhao0813@163.com; Prof. Weihong Qiao: [qiaoweihong@dlut.edu.cn](mailto:qiaoweihong@dlut.edu.cn)

**Figure S1.** Chemical structure of the drug chlorhexidine.

**Figure S2.** SEM micrographs of (a) HM-HAP@CHD, (b) PEG-1000/HM-HAP@CHD, (c) PEG-2000/HM-HAP, (d) PEG-4.6k/HM-HAP, (e) PEI-1800/HM-HAP, (f) PEI-1200/HM-HAP.

**Table S1.** BET surface area, pore size and pore volume of unmodified and polymer-modified HM-HAP particles.

| Sample | BET specific surface area (m^2.^g^-1^) | Pore size (nm) |
| --- | --- | --- |
| HM-HAP | 68.719 | 12.331 |
| PEG-1000/HM-HAP | 51.383 | 12.364 |
| PEG-2000/HM-HAP | 52.039 | 12.365 |
| PEG-4.6k/HM-HAP | 55.152 | 17.281 |
| PEI-1800/HM-HAP | 45.343 | 9.557 |
| PEI-1200/HM-HAP | 36.846 | 9.521 |

**Validation of Beer-Lambert law**

As demonstrated in Fig. S2, the Beer-Lambert law was used to validate the linearity of the UV-Vis response at 254 nm for CHD.

**Figure S3.** UV spectra of CHD at different concentration (a), and Beer-Lambert law validation for CHD at λ = 254 nm (b).

Solutions of different concentrations were prepared by dilution of a 1000 mg/L stock solution. Each solution’s absorption spectrum was obtained at 254 nm. The graph of absorbance vs concentration yielded an R^2^ value of 0.99, indicating that CHD has a strong linear response. The intensity of absorption rises with CHD concentration due to increased contact between the molecules, as proven by the positive value of R^2^.

The graph in Figure S4(a) shows the gel’s modulus (Pa) as a function of strain (%), highlighting two primary parameters i.e., the storage modulus (G′) and the loss modulus (G″). The G′ value indicates the gel’s elastic characteristics, demonstrating that at low strains, the gel primarily functions as a solid, exhibiting significant resistance to deformation. As the strain increase, G′ attains an equilibrium point, signifying that the gel’s structure stabilizes and becomes less prone to further alterations under stress. On the other hand, G″, indicative of the gel’s viscous properties, demonstrates a notable increase at elevated strain levels, implying that the gel starts to display more liquid-like properties as it undergoes deformation. The alteration in rheological behaviour is crucial for knowing the gel’s functionality in vivo, particularly regarding its ability to load and release the chlorhexidine medication. The equilibrium between these elastic and viscous characteristics is essential for the gel’s efficacy in regulated medication delivery to the teeth, assuring that the material retains its shape and distributes the medicine appropriately throughout application.

Figure S4(b) shows the frequency-dependent rheological properties of the PAAS gel to further assess its behavior under different oscillatory stresses. The graph presents the correlation between the storage modulus (G′) and the loss modulus (G″) as functions of frequency (rad/s). The storage modulus (G′) increases with frequency, showing that the gel attains greater stiffness and demonstrates enhanced solid-like characteristics at elevated frequencies. This indicates that the gel exhibits greater resistance to deformation under rapid oscillations, which is crucial for its stability during dynamic situations, such as mastication. Similarly, the loss modulus (G″), also increases with frequency, although at a slower rate relative to G′, showing that the gel’s viscous (liquid-like) characteristics are less prominent under higher-frequency stresses.

**Figure S4.** Rheological properties of PAAS gel (a) Strain sweep test, changes in loss modulus and storage modulus of PAAS gel under 1 Hz conditions (b) Frequency sweep test, changes in loss modulus and storage modulus of PAAS gel under 1% strain conditions.

**Figure S5.** FTIR spectra of gel incorporated polymer modified HM-HAP samples. FTIR spectra of 1% (a), and 5% (b) CHD-loaded polymer-modified HAP incorporated into sodium polyacrylate (PAAS) gels, confirming the successful integration of CHD-loaded HAP into the gel matrix.

The SEM image (Fig. S5) clearly shows the successful integration of the HAP particles within the gel structure, with distinct particle morphology retained, and the gel matrix visible surrounding the particles.

**Figure S6.** SEM images of chlorhexidine-loaded HM-HAP particles incorporated into a sodium polyacrylate (PAAS) gel matrix.

**Figure S7.** SEM micrographs of chlorhexidine-loaded polymer-modified HM-HAP particles incorporated into the sodium polyacrylate (PAAS) gel matrix.

**Figure S8.** Evaluation of antibacterial activity using disc method against (a) *E.coli* (1:PBS, 2:CHD, 3:Gel), (b) *E.coli* (1% CHD-loaded polymer modified HM-HAP gel samples), and (c) *E.coli* (5% CHD-loaded polymer modified HM-HAP gel samples).
